# Successful Experience of Laparoscopic Pancreaticoduodenectomy and Digestive Tract Reconstruction With Minimized Complications Rate by 14 Case Reports

**DOI:** 10.1097/MD.0000000000003167

**Published:** 2016-04-29

**Authors:** Yong Fan, Yanhui Zhao, Lan Pang, Yingxing Kang, Boxiong Kang, Yongyong Liu, Jie Fu, Bowei Xia, Chen Wang, Youcheng Zhang

**Affiliations:** From the Minimally Invasive Surgery Center, The Second Hospital of Lanzhou University, Lanzhou, Gansu Province, China.

## Abstract

Laparoscopic pancreatic surgery is one of the most sophisticated and advanced applications of laparoscopy in the current surgical practice. The adoption of laparoscopic pancreaticoduodenectomy (LPD) has been relatively slow due to the technical challenges. The aim of this study is to review and characterize our successful LPD experiences in patients with distal bile duct carcinoma, periampullary adenocarcinoma, pancreas head cancer, and duodenal cancer and evaluate the clinical outcomes of LPD for its potential in oncologic surgery applications.

We retrospectively analyzed the clinical data from 14 patients who underwent LPD from August 2013 to February 2015 in our institute.

We presented our LPD experience with no cases converted to open surgery in all 14 cases, which included 10 cases of laparoscopic digestive tract reconstruction and 4 cases of open digestive tract reconstructions. There were no deaths during the perioperative period and no case of gastric emptying disorder or postoperative bleeding. The other clinical indexes were comparable to or better than open surgery.

Based on our experience, LPD could be potentially safe and feasible for the treatment of early pancreas head cancer, distal bile duct carcinoma, periampullary adenocarcinoma, and duodenal cancer. The master of LPD procedure requires technical expertise but it can be accomplished with a short learning curve.

## INTRODUCTION

Laparoscopic surgery has gained an increasingly important role in surgical procedures as a result of improvements in laparoscopic equipment, instruments, and surgical techniques. In some areas, open surgery has been replaced by the minimally invasive laparoscopic surgery as the preferred surgical approach.^1^ However, many surgeons do not prefer to perform laparoscopic pancreatic surgery and digestive tract reconstruction owing to its complexity and high incidence of complications. As a result, laparoscopic pancreaticoduodenectomy (LPD) is less widely adopted than other laparoscopic procedures and it is rare for medical institutions to perform LPD surgery on a large scale.^2^ Primary goals for surgeons remain to secure radical resections and to ensure successful digestive tract reconstruction and rehabilitation of patients.

Generally, there are several key step stone factors before the successful completion of LPD. The first factor is the patient selection for surgical procedures. LPD is currently considered suitable for patients with cholangiocarcinoma of the lower segment, periampullary cancer, duodenal cancer, and small diameter pancreas head carcinoma.^3^ Second, a complete set of laparoscopic equipment and instruments is required. These instruments include an ultrasonic knife, BiCision, and Endo-GIA with acceptable cutting and hemostatic effects. Each of these instruments has a different purpose and is necessary for surgery.^4^ Skilled laparoscopic surgical techniques are also required. LPD requires a skilled surgeon with a mastery of laparoscopic suturing and knotting techniques. In addition, the surgeon must have considerable patience and stamina. The surgeon must also have refined open surgery skills and familiarity with the anatomical knowledge.

In this study, we retrospectively reviewed our successful experiences of LPD procedures with digestive tract reconstruction and achieved minimal complication rates in 14 patients. We also aimed to characterize our experience in detail and help surgeons to overcome the initial learning curve for LPD.

## METHODS

### Patients

Between August 2013 and January 2015, LPD procedure was performed on 14 patients consisting of 5 males and 9 females at our institute. The patient demographic characteristics were collected and analyzed as shown in Table 1. Patients’ ages ranged from 42 to 69 years, and the median age was 54 years. All patients had no cardiopulmonary disease or other important organ dysfunction and surgical contraindications. There were 7 cases with periampullary cancer, 5 cases of early pancreas head carcinoma, and 2 cases were lower segment common bile duct cancer. This study was approved by the Institute Ethical Committee of our University, and informed consent was obtained from all the patients for participation in the study.

**TABLE 1:**
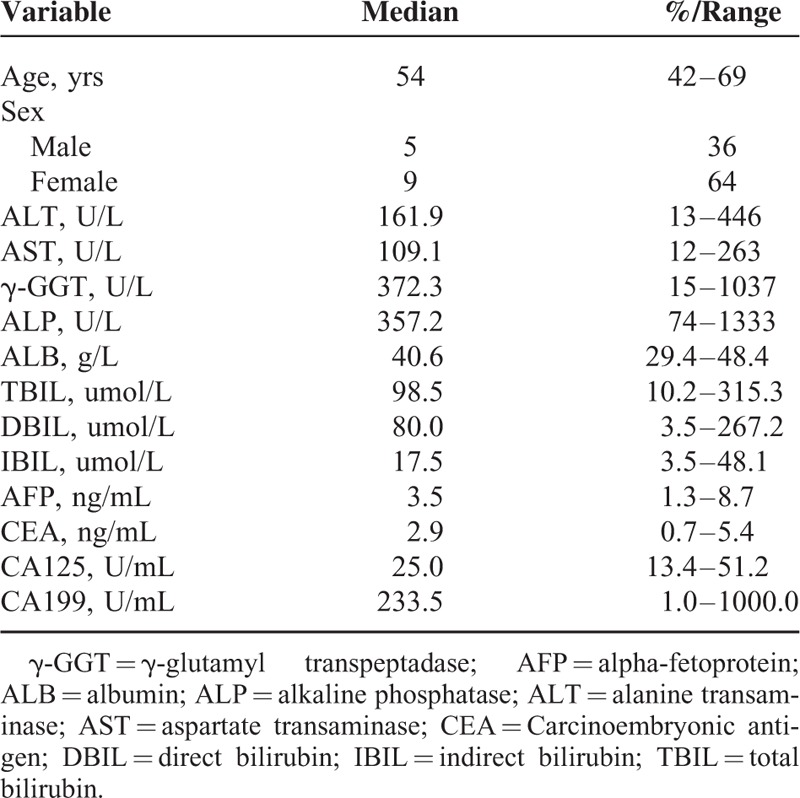
Baseline Characteristics of the Included Patients Underwent Laparoscopic Pancreaticoduodenectomy

### Preoperative Preparation

All patients underwent magnetic resonance cholangiopancreatography or computed tomography scan before surgery to confirm that there was no portal vein, inferior vena cava, or superior mesenteric vascular invasion, and no distant metastasis. There were 10 cases that received endoscopic retrograde cholangiopancretography examination before surgery, and 6 cases received implants in the nasobiliary duct to reduce jaundice. The other 4 cases were given percutaneous transhepatic cholangiodrainage to reduce jaundice. The 4 patients with bilirubin <200 μmol/L did not undergo procedures to reduce jaundice before surgery. The preoperative pathology results confirmed that 9 cases were malignant tumors. Five cases of pancreas head carcinoma were confirmed intraoperatively by frozen pathology examination.

### Position and Operation Incision Location

For each surgery, the patient was positioned supine with 15° of head elevation. The surgeon stood by the right side of the patient. A 10 mm longitudinal incision was made at the navel, and a trocar was inserted into the incision. A trocar was placed at the umbilical level 10 mm to the right of the clavicle midline and was used as the main surgical site. One trocar was also placed at the following locations: the umbilical level of the right anterior axillary line costal margin, the left subclavian and left subclavian midline, and 5 mm from the midline lateral subcostal region.

### Surgical Methods

The liver, bowel, abdominal, and omental were probed in sequence for mestastases. The omentum was flipped onto its head and cut on the transverse colon upper edge to reveal the pancreas (pancreas specimens were obtained for frozen tissue examination). The pancreas serous lower edge was cut to reveal the superior mesenteric vein (SMV). The portal vein was isolated carefully by blunt dissection along the posterior edge of SMV and the neck of pancreas to further determine whether the tumor was excisable.

### Resection of Pancreas and Duodenum

**FIGURE 1:**
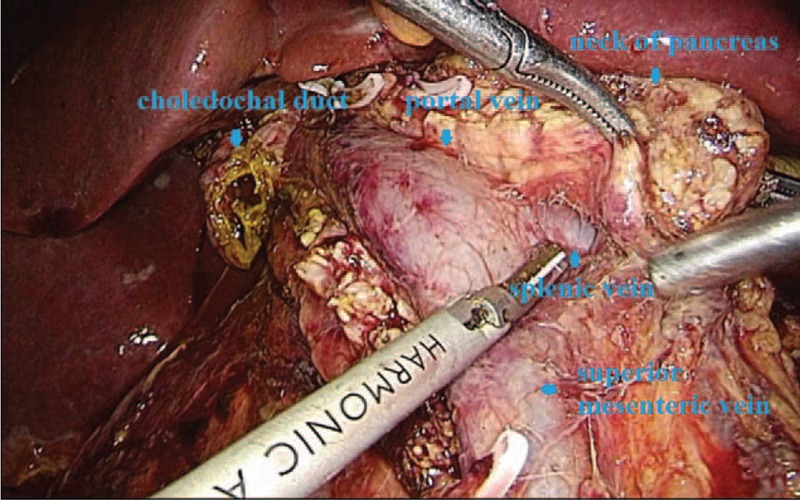
The anatomical exposure of the abdominal wall puncture and specimen removal incision.

The liver-duodenum ligament was cut, and lymph nodes were spared. The common bile duct and hepatic artery were freed while the portal vein was exposed (Figure 1). The right gastric artery was cut to expose the hepatic artery and gastroduodenal artery. The right gastroepiploic artery and vein were cut, and then the distal portion of the stomach was cut with Endo-GIA. The duodenum lateral peritoneum was opened, and the Kocher incision was extended to the level of the horizontal part of the duodenum. The duodenum and pancreatic head were freed along the right kidney front fascia to the inferior mesenteric vein. The duodenum was then pulled to free the horizontal part to the Treitz ligament. The transverse colon was lifted and the proximal jejunum was transected with Endo-GIA 15 cm from the Treitz ligament. The mesentery around the Treitz ligament and proximal jejunum was cut and pulled to the right rear from the superior mesenteric artery. Then, the pancreatic neck was transected along the SMV by an ultrasound scalpel at the main pancreatic duct. The duodenum was stretched to the upper rights to fully expose both the pancreatic head and the uncinate, which were removed upward from the bottom. The pancreatic upper and lower veins were cautiously treated with a biological clamp, and the remaining small branches were cut using an ultrasound knife and safe speed knife. The head of the pancreas and uncinate were completely cut to remove the gallbladder, and the lower part of the common bile duct was transected.

### Digestive Tract Reconstruction

**FIGURE 2:**
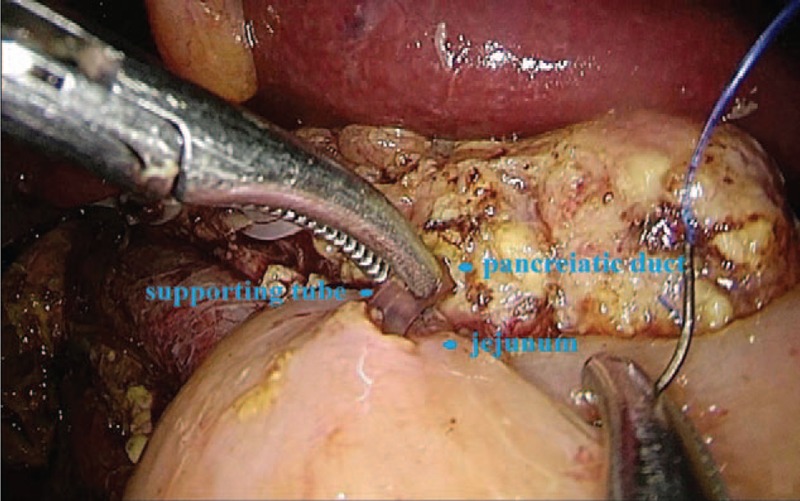
Observation of pancreatic duct-jejunum end-side anastomosis with pancreatic duct-jejunum end-side anastomosis.

**FIGURE 3:**
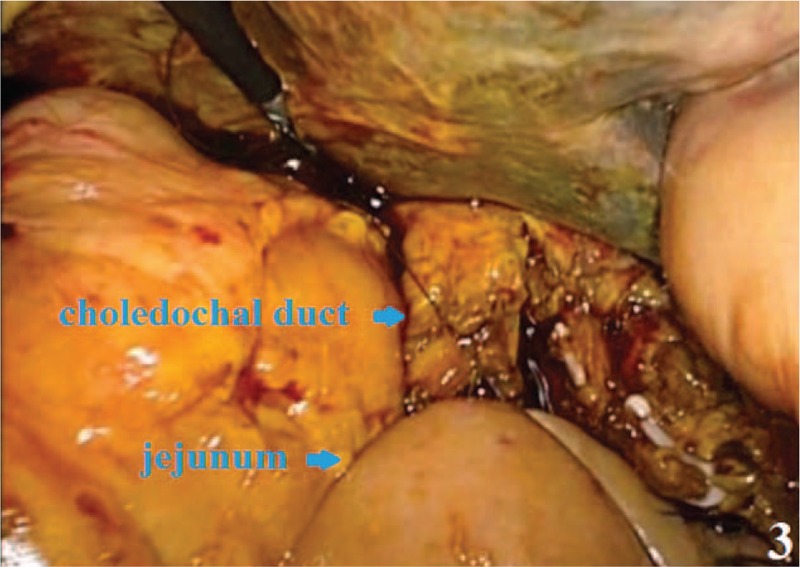
Observation of bile duct-jejunum end-side anastomosis.

**FIGURE 4:**
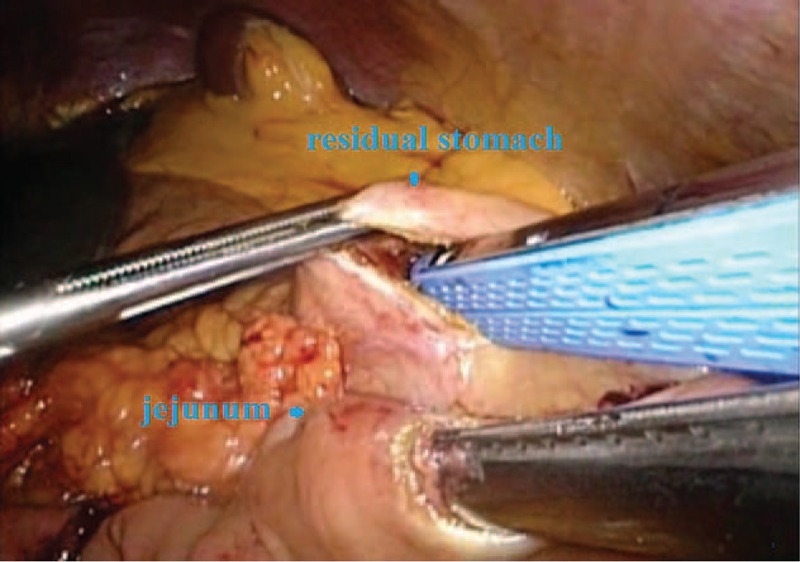
Observation of gastric-jejunum side-side anastomosis.

**FIGURE 5:**
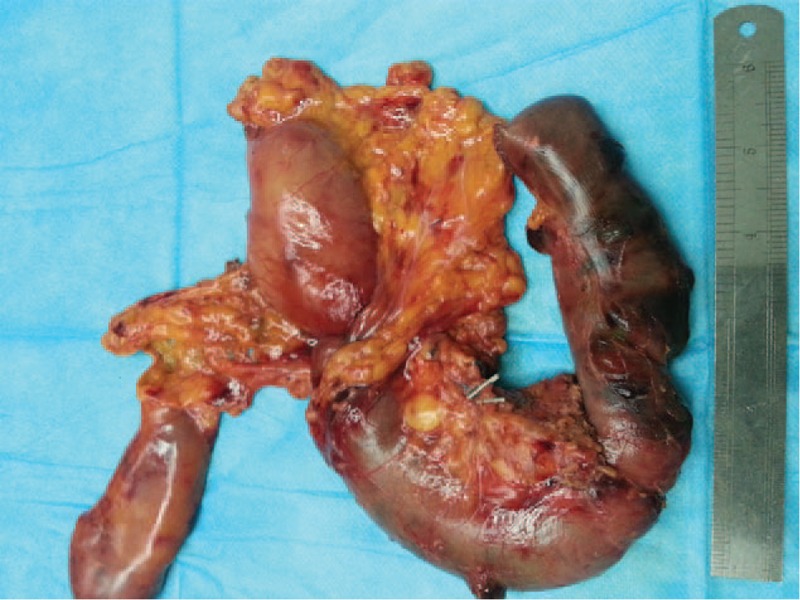
Observation of gross specimen of stomach, pancreas head, common bile duct, and tumor.

**FIGURE 6:**
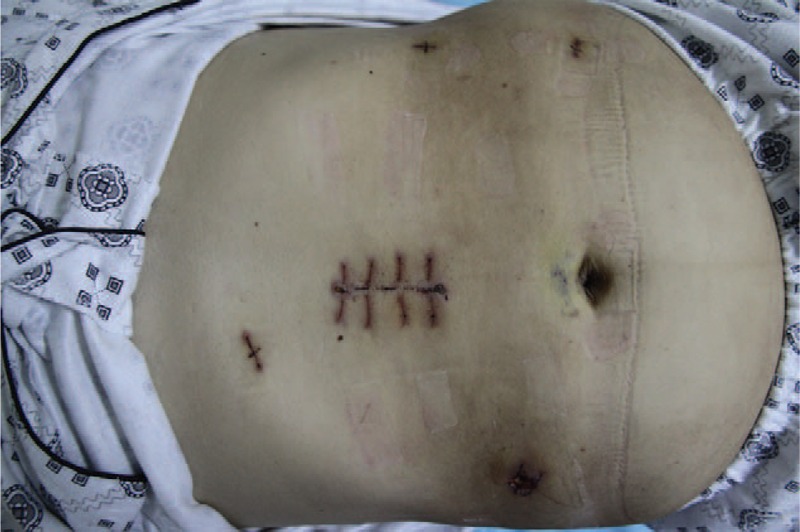
The abdominal wall puncture and specimen removal incision.

A small hole was cut in the mesangial side. The jejunum was lifted 10 cm before the colon stump. The bowel wall and pancreas posterior edge were continuously sutured with 4-0 Prolene. One end of the support tube was placed into the pancreatic duct at the jejunum posterior wall (Figure 2). The pancreatic duct was sutured through all layers with 4-0 Prolene line, which normally requires 3 needles. The other end of the support tube was inserted into the jejunum without fixation. The pancreatic duct-jejunum anterior wall anastomosis was performed with continuous sutures using the aforementioned method. The pancreas anterior edge, bowel wall, and muscularis-sesora layer were continuously sutured, followed by end-side pancreatic duct-jejunum anastomosis. The bile duct-jejunum was closed with continuous sutures using 4-0 Prolene line to complete bile duct-jejunum end-side anastomosis 10 cm from the distal pancreatic duct-jejunum anastomosis (Figure 3). The anterior wall of the remnant stomach was anastomosed with Endo-GIA approximately 50 cm from the distal bile duct-jejunum anastomosis (Figure 4). A Braun anastomosis was performed with Endo-GIA to prohibit bile reflux. This anastomosis was located approximately 20 cm from the distal end and distal jejunum side-by-side anastomosis. Thus, the digestive tract reconstruction was completed. Finally, a 4to 5 cm incision was made in the middle epigastric site to remove specimens. The specimen was then placed into the specimen bag and sent to pathological examinations (Figures 5 and 6).

## RESULTS

A total of 14 patients were successfully treated with laparoscopic pancreas duodenum resection. There were 10 cases of complete laparoscopic digestive tract reconstruction. The other 4 cases received open digestive tract reconstruction via a small incision in the middle epigastric site to shorten the operation time due to poor general condition.

The operative details and postoperative outcomes are summarized in Table 2. The surgical time was 6 to 12.5 hours, and the average was 7.5 hours. The surgical time for the first 5 cases was longer (7.0–12.5 hours) than the latter 9 cases (6–8 hours). The intraoperative hemorrhage was 230 mL to 650 mL, and the average was 310 mL. In 6 cases, intraoperative blood transfusions were given. The patients were aerofluxus in 3 to 5 days postoperatively, and the average was 4.5 days. After aerofluxus the nasogastric tube was removed, the patients were given a liquid diet. The average postoperative eating time was 5 days (4–8 days).

**TABLE 2:**
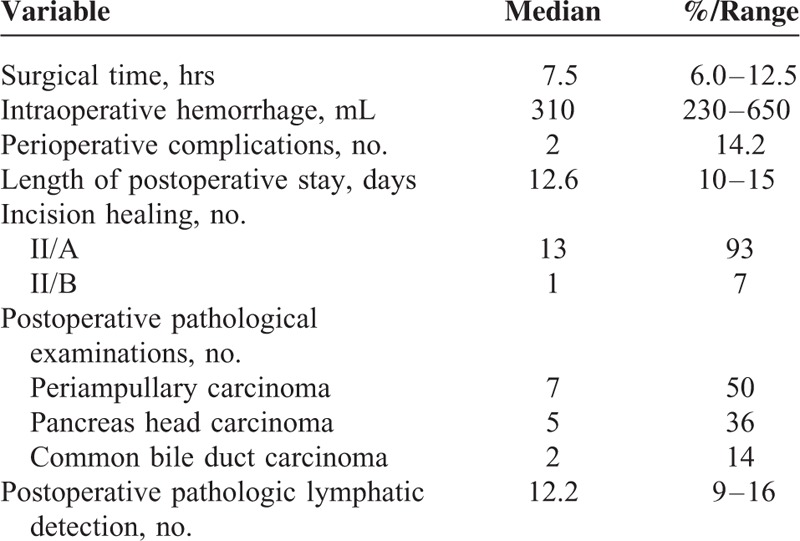
Operative Details and Postoperative Outcomes of the Included Patients Underwent Laparoscopic Pancreaticoduodenectomy

The incidence of perioperative complications was 14.2% (2/14). One case developed a pancreatic fistula and was discharged with a drainage tube after adequate drainage. The patient recovered 2 months later after the drainage tube was withdrawn. The other patient had postoperative bile leakage. The initial leakage was approximately 50 mL daily; 7 days later, the leakage stopped. The other patient was discharged after drainage tube withdrawal. The length of postoperative stay was 10 to 15 days, and the average was 12.6 days. There were no cases of gastric emptying disorder, postoperative hemorrhage, or perioperative death. The postoperative pathological examinations showed 7 cases of periampullary carcinoma, 2 cases of carcinoma of the lower segment of the common bile duct, and 5 cases of early pancreatic head carcinoma. The average postoperative pathologic lymphatic detection was 12.2 (9–16). There were 13 cases of R0 resection. One case of pancreatic head carcinoma received an R1 resection based on postoperative pathological examination.

All 14 cases had postoperative periodic follow-up, and the average follow-up was 5.5 months (2–16 months). There was a retroperitoneal lymph node metastasis found in 1 case of pancreatic cancer 6 months after surgery. The patient survived for 10 months. There were no other patient deaths during follow-up.

## DISCUSSION

Compared with traditional surgery, laparoscopic surgery offers advantages of rapid recovery, fewer trauma, less hemorrhage, and shorter hospitalization.^5^ However, the development of laparoscopic surgery has been slow.^6,7^ The difficulty of adopting laparoscopic pancreatic surgery is possibly due to the complexity of the pancreatic-duodenal anatomical structure. Also, the perfect performance of digestive tract reconstruction requires a skilled laparoscopic suture technique and substantial experiences, which serves as one of the important aims of this study. In this study, we achieved successful LPD experiences in 14 patients with minimized complication rates with several important aspects to be considered for completing pancreatic duodenal laparoscopic resection.

The first aspect is patient selection. In our study, there were 14 cases, and 7 cases were periampullary carcinoma. There were also 2 cases with carcinoma of the lower segment of the common bile duct. The remaining 5 cases were early pancreatic head carcinoma. For the first 2 cases, the dilation to the bile duct and pancreatic duct was obvious because of early jaundice. The confirmation with preoperative pathological examination and the laparoscopic resection with digestive tract reconstruction was relatively easy in those cases. For the patients with pancreatic head cancer, it was difficult to make an early diagnosis because of the invasion into surrounding tissues, and potential distant metastases. Also, the dilation of the bile duct and pancreatic duct was not obvious. A mass >3 cm was difficult to remove, and the digestive tract reconstruction was therefore more complex. Thus, our experiences suggested that laparoscopic pancreatic duodenal resection is more suitable for periampullary cancer, lower bile duct cancer, and pancreatic head carcinoma with a mass <3 cm. This view is also consistent with other researchers.^8^

With respect to the surgical approach, our experiences suggested that cutting the gastrocolic ligament to expose the lower edge of the pancreas and the SMV and then separating the portal vein is beneficial for determining whether the tumor can be removed. In this study, an extension Kocher incision was used on the horizontal part of the duodenum, and the site behind the duodenum and pancreas uncinatus was fully freed. The hepatoduodenal ligament lymph nodes were clear, and the common bile duct was freed (the bile duct was not cut). After the distal stomach was cut, the pancreas neck was cut along the SMV and portal vein (with care taken to find and mark the pancreatic duct). After the jejunum was cut, it was pulled to the right behind the mesenteric vessel. The pancreatic head was pulled to the upper right, and the pancreas uncinatus was resected from the bottom up to the lower common bile duct. The hepatoduodenal ligament lymph nodes were cleared after resection of the gallbladder. The common bile duct was cut to excise the whole piece of the gallbladder. There are some important suggestions for this surgery from the authors. First, be gentle. Excessive pull may cause venous branch tearing, and the surgery will not be able to proceed due to obstructed visual fields. Second, the coarser vascular branch should be blocked with biological clamps, and 5 mm small biological clamps are preferred because they are convenient and flexible. Third, the resection of pancreas uncinatus should be complete; avoid damaging the superior mesenteric artery.^9^

Useful and substantial experience was obtained in reconstructing the digestive tract. First, in the pancreatic duct-jejunum end-side anastomosis, the jejunum openings should be close to the front bowel wall and should be sutured with 4-0 Prolene line to protect the pancreas from damage. A supporting tube is necessary for assisting the mucous membrane suture. Second, in the bile duct-jejunum anastomosis, the jejunum opening is close to the front of the duct. Thus, stitching of the back wall should be performed from left to right. After knotting on the right side, the anterior wall should be sutured from right to left. Third, compared with the stomach posterior wall, the gastric anterior wall-jejunum anastomosis with Endo-GIA is simple. Suturing anastomosis with a barbed line is safe and fast.^10,11^ Adding a Braun anastomosis can effectively prevent bile reflux and anastomotic fistula.

By minimizing surgical complication incidences, patient rehabilitation is improved, which is the primary goal of the surgeon. Among our 14 cases, there were only 2 postoperative complications, which is significantly fewer than previous reports.^6^ The following reasons could be responsible for the low complication rates. First, the case selection was performed relatively cautiously, and the patients had early cancer and acceptable general health. Second, we performed meticulous operative technique to minimize the unnecessary damage and accidental hemorrhage. The digestive tract reconstruction method was especially important. High-quality stitching and a skilled laparoscopic suture technique avoided the occurrence of postoperative anastomotic fistulas. The percentage of complications after open pancreas duodenum resection, such as total postoperative complications, pancreatic fistula, biliary leakage, anastomotic leakage and abdominal infection, gastrointestinal emptying dysfunction, and anastomotic hemorrhage, was not significantly different from the complications after LPD.^3^

In conclusion, we demonstrated that LPD could achieve similar perioperative outcomes as open surgery for patients with early pancreas head cancer, distal bile duct carcinoma, periampullary adenocarcinoma, and duodenal cancer. In addition, patients may potentially suffer from fewer trauma, and result shorter healing time. Furthermore, the surgical complications such as incision bleeding and infection can be minimized, and the postoperative recovery could be fast. This procedure does require technical skills to ensure good results as described in this article, but it is applicable and proficiency can be achieved with a short learning curve following our experience.

## References

[1] Evangelista S Drug evaluation: Pumosetrag for the treatment of irritable bowel syndrome and gastroesophageal reflux disease. *Curr Opin Investig Drugs* 2007; 8:416–422. 17520871

[2] Mesleh MG Stauffer JA Bowers SP Cost analysis of open and laparoscopic pancreaticoduodenectomy: a single institution comparison. *Surg Endosc* 2013; 27:4518–4523. 23943116 10.1007/s00464-013-3101-6

[3] Asbun HJ Stauffer JA Laparoscopic vs open pancreaticoduodenectomy: overall outcomes and severity of complications using the Accordion Severity Grading System. *J Am Coll Surg* 2012; 215:810–819. 22999327 10.1016/j.jamcollsurg.2012.08.006

[4] Boggi U Signori S De Lio N Feasibility of robotic pancreaticoduodenectomy. *Br J Surg* 2013; 100:917–925. 23640668 10.1002/bjs.9135

[5] Kendrick ML Cusati D Total laparoscopic pancreaticoduodenectomy: feasibility and outcome in an early experience. *Arch Surg* 2010; 145:19–23. 20083750 10.1001/archsurg.2009.243

[6] Boggi U Amorese G Vistoli F Laparoscopic pancreaticoduodenectomy: a systematic literature review. *Surg Endosc* 2015; 29:9–23. 25125092 10.1007/s00464-014-3670-z

[7] Jacobs MJ Kamyab A Total laparoscopic pancreaticoduodenectomy. *JSLS* 2013; 17:188–193. 23925010 10.4293/108680813X13654754534792 PMC3771783

[8] Tamura A Otsuka Y Tsuchiya M Laparoscopic surgery for pancreatic cancer. *Gan To Kagaku Ryoho* 2012; 39:351–356. 22421760

[9] Ogiso S Conrad C Araki K Posterior approach for laparoscopic pancreaticoduodenectomy to prevent replaced hepatic artery injury. *Ann Surg Oncol* 2013; 20:3120. 23793363 10.1245/s10434-013-3058-7

[10] Cho A Yamamoto H Kainuma O Tips of laparoscopic pancreaticoduodenectomy: superior mesenteric artery first approach (with video). *J Hepatobiliary Pancreatic Sci* 2014; 21:E19–21. 10.1002/jhbp.54 24307512

[11] Corcione F Pirozzi F Cuccurullo D Laparoscopic pancreaticoduodenectomy: experience of 22 cases. *Surg Endosc* 2013; 27:2131–2136. 23355144 10.1007/s00464-012-2728-z

